# Circulating Cell-Free DNA in Liquid Biopsies as Potential Biomarker for Bladder Cancer: A Systematic Review

**DOI:** 10.3390/cancers13061448

**Published:** 2021-03-22

**Authors:** Raquel Herranz, Julia Oto, Emma Plana, Álvaro Fernández-Pardo, Fernando Cana, Manuel Martínez-Sarmiento, César D. Vera-Donoso, Francisco España, Pilar Medina

**Affiliations:** 1 Haemostasis, Thrombosis, Arteriosclerosis and Vascular Biology Research Group, Medical Research Institute Hospital La Fe, Avenida Fernando Abril Martorell 106, 46026 Valencia, Spain; raq.herranz@gmail.com (R.H.); juliaotomartinez@gmail.com (J.O.); plana_emm@gva.es (E.P.); alvarofernandezpardo@gmail.com (Á.F.-P.); fernandocana1998@gmail.com (F.C.); espanya_fra@gva.es (F.E.); 2 Angiology and Vascular Surgery Service, La Fe University and Polytechnic Hospital, Avenida Fernando Abril Martorell 106, 46026 Valencia, Spain; 3 Department of Urology, La Fe University and Polytechnic Hospital, Avenida Fernando Abril Martorell 106, 46026 Valencia, Spain; mmarsar@gmail.com (M.M.-S.); cdveradonoso@gmail.com (C.D.V.-D.)

**Keywords:** bladder cancer, blood, cell-free DNA, early diagnosis, liquid biopsy, noninvasive, predictive markers, prognosis, tumor biomarkers, urine

## Abstract

**Simple Summary:**

Bladder cancer (BC) is one of the most frequent cancers in the world and the urological tumor that presents the highest mortality. The diagnostic and prognostic methods available at present for BC are expensive and highly invasive for the patient, so the pursue of biomarkers that may replace those methods has been ongoing for years with limited success. One of these potential biomarkers is cell-free DNA, which can be found in liquid biopsies such as urine and blood. The present review summarizes the most recent research findings in the study of cell-free DNA to diagnose BC and even monitor treatment.

**Abstract:**

Bladder cancer (BC) is among the most frequent cancer types in the world and is the most lethal urological malignancy. Presently, diagnostic and follow-up methods for BC are expensive and invasive. Thus, the identification of novel predictive biomarkers for diagnosis, progression, and prognosis of BC is of paramount importance. To date, several studies have evidenced that cell-free DNA (cfDNA) found in liquid biopsies such as blood and urine may play a role in the particular scenario of urologic tumors, and its analysis may improve BC diagnosis report about cancer progression or even evaluate the effectiveness of a specific treatment or anticipate whether a treatment would be useful for a specific patient depending on the tumor characteristics. In the present review, we have summarized the up-to-date studies evaluating the value of cfDNA as potential diagnostic, prognostic, or monitoring biomarker for BC in several biofluids.

## 1. Introduction

Bladder cancer (BC) is the 12th most frequent cancer worldwide, ranking 14th regarding mortality from cancer per year [1]. In 2018, the incidence of BC was 549,393 new cases per year and provoked 199,922 deaths. BC is responsible for 3% of all malignant tumors in adults and is the most lethal urological malignancy [1,2]. The contribution of BC to trends in medical expenses is significant as, by the end of the decade, it is expected to account for >3% of all cancer-related medical costs. In fact, BC is the most expensive cancer to treat, with the cost of muscle-invasive BC (MIBC) approaching $150,000 per capita [3,4].

**Figure 1:**
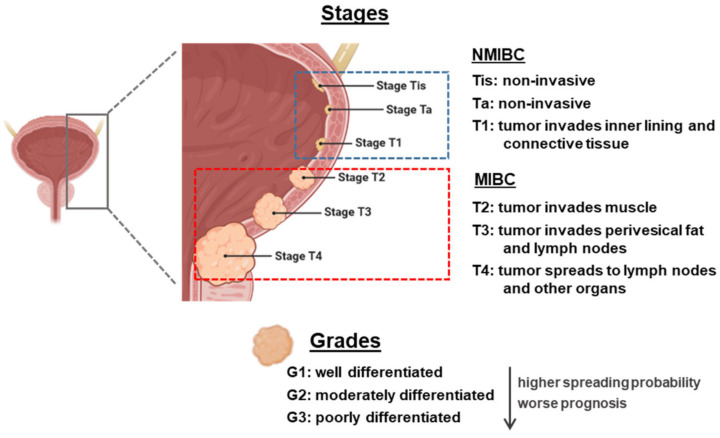
Overview of staging and grading of bladder cancer tumor. The figure was created with BioRender.com. NMIBC: non-muscle-invasive bladder cancer; MIBC: muscle-invasive bladder cancer.

From an anatomopathological point of view, the classification of BC is made with two parameters: grading and staging. There is a clear correlation between the grade and stage and the prognosis and outcome of BC patients (Figure 1). Grading indicates the degree of cellular differentiation of the tumor. It is a measurement of aggressiveness. The grade of a cancer is usually described using a number system ranging from G1 to G3 (being high-grade cancers more likely to spread and more poorly differentiated, thus very different from normal bladder cells) [5].

Staging indicates how far a cancer has spread, with the lower-stage cancers being smaller and with better prognosis regarding treatment outcomes. The most widely used staging system for BC is known as the TNM system (tumor, lymph nodes, and metastasis). There are two subdivisions: non-muscle-invasive BC (NMIBC) that can be staged as Tis (flat tumor or carcinoma in situ, with cancer cells only in the inner layer of the bladder lining or urothelium), Ta (papillary cancer, only in the inner layer of the bladder lining), or T1 (when the tumor has started growing into the layer of connective tissue, beneath the bladder lining), or MIBC, ranging from T2 to T4 (the bigger the number, the bigger the level of infiltration). N stands for the degree of invasion into nearby lymph nodes, and ranges from N0 to N3. M stands for metastasis, being M0 tumors who have not invaded other body organs and M1 those who have [5,6].

Among BC patients, 70% present NMIBC, formerly known as superficial BC (this term should no longer be used as it is incorrect), and the remainder present MIBC, known as invasive BC. In NMIBC patients, 70% present Ta lesions, 20% T1 lesions, and 10% Tis [7]. These tumors can be treated by endoscopic transurethral resection of the bladder (TURB), eventually in combination with intravesical instillations. MIBC is a heterogeneous, aggressive disease, associated with a 5-year survival rate of 60% for patients with localized disease and <10% for patients with distant metastases, and is characterized by genomic instability and a high mutation rate [8].

Most BCs are diagnosed after patients experience macroscopic hematuria. Currently, cytology and cystoscopy are the gold standards for BC detection. Cytology, consisting in checking urine under a microscope for abnormal cells, is a noninvasive test that can identify tumor cells shed in urine but presents low sensitivity, especially in low-grade tumors. Cystoscopy consists in the insertion of a cystoscope, a thin tube-like instrument with a light and a lens for viewing, through the urethra, to observe the inner wall of the bladder on a computer monitor. It allows checking for abnormal areas and even to perform transurethral resection of bladder tumor, which can be used to confirm BC diagnosis by deep visualization of the bladder and muscle (if possible) and also serves as the first stage of treatment. However, cystoscopy is a highly invasive examination that can also cause pain and other complications [6,9,10,11].

When those techniques give rise to suspicion of invasive BC or the patient is at high risk of developing invasive BC, other techniques are used for staging, such as computerized tomography, magnetic resonance imaging or even positron emission tomography scan [9,10]. The lymph nodes are a common site of metastatic spread in patients with BC; thus, these techniques are employed for preoperative nodal staging by detecting malignant lymph nodes based on size. However, normal-sized or minimally enlarged lymph nodes represent a considerable proportion of malignant lymph nodes in BC patients, which leads to under staging patients with small nodal metastases. Consequently, the sensitivity of computerized tomography or magnetic resonance imaging for detecting malignant lymph nodes in BC is relatively low (31–45%) [12].

Although urine cytology is the easiest noninvasive test that shows a high specificity, it shows a low sensitivity especially for low-grade BC tumors and a low degree of reproducibility [13]. To overcome these drawbacks, a large number of Food and Drug Administration (FDA)-approved urinary tests have been proposed such as Bladder Tumor Antigen (BTA) stat and BTA TRAK (Polymedco Inc., Cortlandt, NY, USA), NMP22 kit and NMP22 BladderChek (Alere, Waltham, MA, USA), ImmunoCyt/uCyt+ assay (Scimedx Inc., Denville, NJ, USA), and UroVysion (Vysis, Abott Molecular Inc., Chicago, IL, USA) [14]. However, none have been implemented in daily clinical practice to replace cystoscopy as they fail to diagnose 18–43% of BCs and yield a 12–26% false positive rate [14]. Accordingly, novel noninvasive markers able to ascertain the absence of tumor would be of utmost importance for clinicians because the first objective is to avoid unnecessary cystoscopies.

Liquid biopsy refers to the analysis tool that provides the opportunity to detect, analyze and monitor cancer and other diseases by using biomarkers, which allows a lower invasiveness than using tissue biopsies. The most commonly analyzed biofluids are plasma, serum, urine, saliva, breast milk, or cerebrospinal fluid, in which it is possible to evaluate markers to monitor tumor heterogeneity [15]. Liquid biopsies provide many advantages over the usage of conventional tissue biopsies: obtaining tissue biopsies requires surgical intervention and its accessibility could be an issue depending on tumor location and its heterogeneity, as intra-tumor heterogeneity may lead to unreliable results. Furthermore, monitoring tumor progression and patient evolution with tumor biopsies is unfeasible, so minimally invasive procedures are in strong need. Accordingly, liquid biopsy represents an ideal procedure for such applications, since it may solve all the aforementioned problems: it is easy to obtain, minimally invasive or even noninvasive, applicable to serial monitoring, allows the evaluation of tumor progression and it is representative of the whole tumor tissue, thus circumventing the drawback of tumor heterogeneity [16,17].

**Figure 2:**
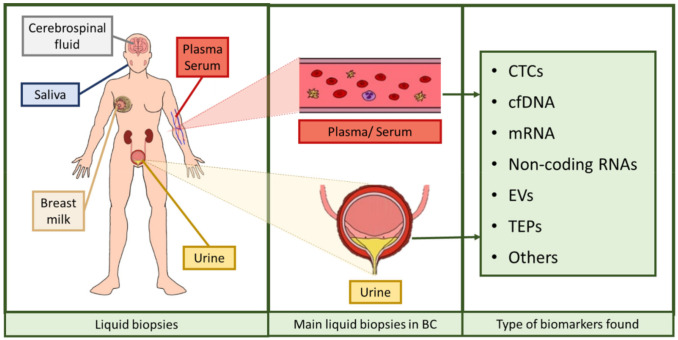
Assortment of liquid biopsies and biomarkers found in them. Liquid biopsies comprise serum, plasma, urine, breast milk, saliva, and cerebrospinal fluid, among others. In BC, the most used liquid biopsies are serum, plasma, and urine. The biomarkers comprised in these liquid biopsies are circulating tumor cells (CTCs), cell-free DNA (cfDNA), messenger RNA (mRNA), several types of non-coding RNAs, extracellular vesicles (EVs), and tumor-educated platelets (TEPs), among others [16,18]. The figure was created using Krita.

Over the last years, research has been focused on the identification of noninvasive biomarkers from liquid biopsies. Circulating tumor cells (CTCs), circulating cell-free nucleic acids (DNA, messenger RNA (mRNA), non-coding RNAs such as microRNAs or long non-coding RNAs), tumor-educated platelets (TEPs), or extracellular vesicles (EVs) [16,18] are novel potential biomarkers identified (Figure 2). The term circulating cell-free DNA (cfDNA) refers to fragmented DNA found in the non-cellular components of liquid biopsies, with blood and urine being the most studied biofluids [19]. This fragmented DNA is released after apoptosis and necrosis of both healthy and tumor cells, and it is usually found as double-stranded fragments [19,20,21,22]. Those cfDNA molecules were discovered in 1948 but considered clinically irrelevant until higher levels of cfDNA were evidenced in cancer patients compared to healthy individuals, as a fraction of that cfDNA is released by cancer cells, the so-called circulating tumor DNA (ctDNA). This ctDNA has a unique genetic and epigenetic pattern that mirrors that of the tumor source [21,22,23,24], and the load of this DNA seems to correlate with tumor staging and prognosis [25]. In such conditions of tissue stress, the amount of cfDNA found in biofluids increases, both as small fragments and large fragments [26,27,28,29]. These are thought to be mainly originated from cell apoptosis and necrosis, respectively, and partially by other mechanisms such as autophagy or mitotic catastrophe, although additional studies are needed to ascertain their role [26,27,29,30,31]. In addition, another type of DNA may also be valuable when assessing a disorder with liquid biopsy. Urinary cfDNA originates either from shedding genitourinary tract cells into urine or from cfDNA in circulation passing through glomerular filtration. The latter is also known as transrenal DNA (trDNA) [32] and is originated from cells dying throughout the body. DNA is released by apoptotic and necrotic cells into plasma. A portion of these fragments cross the kidney barrier and appear in urine in the form of 150–200 bp fragments [33,34]. Thus, trDNA has been previously proposed as a source of biomarkers for diagnosis and disease progression monitoring in cancer [28,35].

Accordingly, the analysis of cfDNA, or even that of ctDNA and trDNA, in liquid biopsies reveals as a promising marker for diagnosis, staging, prognosis, monitoring progression, and response to treatment and even for identifying acquired drug resistance in cancer patients. Thus, herein we focused on the role of cfDNA as potential biomarker for BC.

**Figure 3:**
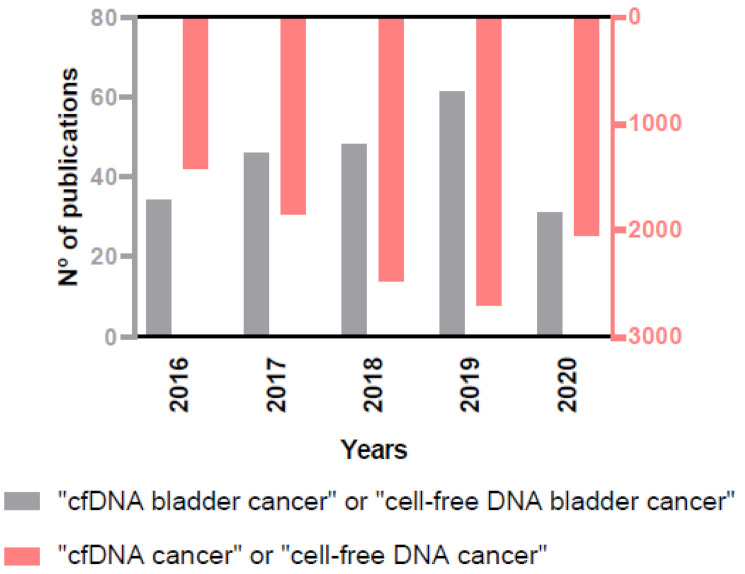
Increasing number of annual publications on the role of cfDNA in cancer and in BC in the PubMed database.

The importance of the role of cfDNA as biomarker in cancer, and specifically in BC, is reflected in the rising number of annual publications on this issue in the PubMed database (Figure 3), and that is the reason why we decided to provide an up-to-date view on the clinical scope of cfDNA analysis in BC.

## 2. Materials and Methods

**Figure 4:**
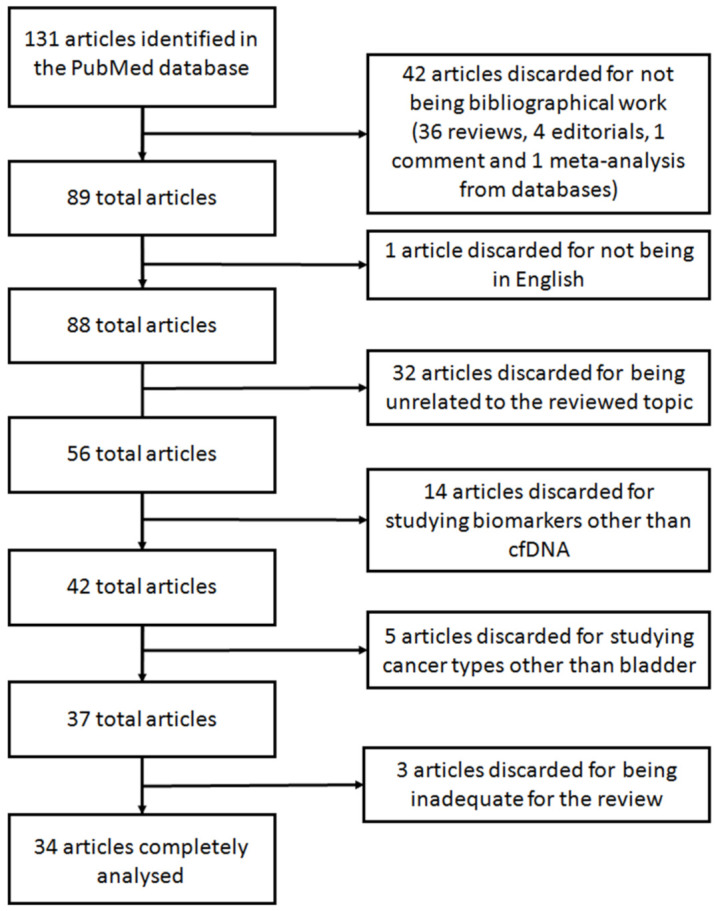
Flowchart showing the selection of articles for the systematic review.

A systematic search was carried out using the database PubMed between the 5th and the 27th of November 2020. The search was performed using the terms “cfDNA bladder cancer” (75 results) and “cell-free DNA bladder cancer” (343 results). When the search was performed using the equivalent Medical Subject Headings (MeSH) terms (“Urinary Bladder Neoplasms”[Mesh]) AND “Cell-Free Nucleic Acids”[Mesh], only 40 results were retrieved. Of these, 17 articles referred to other types of nucleic acids (e.g., RNA, miRNAs, and lncRNAs) and were invalid for our review. This limited number of articles retrieved may be caused because not all articles included in PubMed have been indexed with MeSH terms. Accordingly, we decided to further analyze the comprehensive set of articles rendered by the search “cfDNA bladder cancer” and “cell-free DNA bladder cancer”. Given that the search rendered a vast increase on articles in the last 5 years, we limited the search to this time frame. Thus, a total of 131 eligible articles were found. From these, only the articles studying cfDNA derived from liquid biopsies in BC were further analyzed. Accordingly, articles studying other biomolecules from liquid biopsies such as miRNA, extracellular vesicles or DNA contained in those, long non-coding RNAs or CTCs or articles regarding other cancer types, such as renal or lung cancer, were excluded. Figure 4 shows the flowchart with the selection of the articles reviewed. Forty-two articles were discarded for not being original work: 36 reviews, four editorials, one commentary, and one meta-analysis from databases. Another article was discarded for being in Chinese. Other 32 articles were discarded for being unrelated to the reviewed topic. Fourteen additional articles were discarded for studying biomarker other than cfDNA. Five articles were also discarded for studying cancer types other than bladder. After deep analyzing the remaining 37 studies, three additional articles were inadequate for the purpose of this review for studying a disease different than cancer, for studying genomic DNA instead of cfDNA and for analyzing genomic information from a public database, and thus were discarded. Consequently, the description of the remaining 34 articles is thereafter discussed.

Additionally, Table S1 details information about the number of patients enrolled in each study, the type of liquid biopsy employed for the purpose of each study, the volume of starting material, the cfDNA isolation method used, the detection method, the genes studied, and also the clinically relevant findings obtained in each study reviewed.

## 3. Results and Discussion

### 3.1. Technical Aspects in the Study of cfDNA from Liquid Biopsies

#### 3.1.1. cfDNA Collection

Several studies have focused on developing special tubes that may keep cfDNA intact in samples and uncontaminated from cellular DNA that may be released during centrifugation. For example, to collect whole blood, even though EDTA tubes are the most commonly used, cell-free DNA blood collection (Streck, La Vista, NE, USA) tubes seem to be a better option, as they contain a proprietary formaldehyde-free preservative that stabilizes white blood cells, preventing the release of genomic DNA and allowing isolation of high quality cfDNA [36]. In case of urine collection, standardized urine collection tubes were developed to prevent urine cfDNA degradation and to maintain urine cells in their original form during sample collection, ensuring stabilization of the original proportion and integrity of urine cfDNA, and also minimizing the background noise that may be caused by urinary cellular DNA releasing [37]. Sample centrifugation is also a matter of discussion among studies, being a critical step to avoid cellular genomic DNA contamination. Thus, it is of paramount importance to standardize such pre-analytical conditions as they can highly affect the results of the experiments [24,38,39].

These observations reveal that additional efforts are needed to stablish the best pre-analytical conditions for cfDNA studies in BC to avoid discrepancies among different laboratories and, thus, achieve a precise clinical assessment.

#### 3.1.2. cfDNA Isolation

Presently, no standardized method exists to isolate cfDNA from liquid biopsies. Table S1 summarizes the different methods for cfDNA isolation used when studying cfDNA in BC. Some companies have developed kits that enable the isolation of cfDNA from blood or urine. Although the most used is the QIAamp Circulating Nucleic Acid Kit (QIAGEN, Hilden, Germany), no consensus exists on the most optimal for it (see Table S1 for more kits used for cfDNA isolation). Furthermore, other techniques like the ion-tagged oligonucleotides-magnetic ionic liquid (ITO-MIL) technique, which selectively and rapidly pre-concentrates cfDNA from diluted plasma [40], are under development to isolate cfDNA from liquid biopsies.

Future studies are needed to ascertain the best isolation method for cfDNA from urine to obtain reproducible inter-laboratory results.

### 3.2. Analysis of cfDNA as Diagnostic and Prognostic Biomarker for BC

A first matter of debate is the origin of the best source of urine DNA for BC diagnosis. cfDNA can be studied after cell debris elimination by centrifugation or DNA from this cellular fraction can also be evaluated. Somatic mutations are reliably detected in both urinary cell pellet DNA and cfDNA, and it seems to be comparable to those found in tumor tissue [41]. Additionally, Togneri et al. [42] observed that cfDNA seemed to have higher analytical sensitivity for detection of clinically actionable genomic aberrations than cellular DNA. However, in the particular analysis, two abundant point mutations in the *TERT* promoter (228C>T/250C>T) seemed to be more abundant in cellular DNA than in cfDNA [43]. Thus, the advantage of cfDNA analysis for common BC mutations remains a matter of debate. Hentschel et al. [44] focused on evaluating methylation markers in different urine fractions (cfDNA, pellet and full void urine) compared to paired tumor specimens. For most markers studied they evidenced a correlation in all urine fractions, observing the highest correlation in methylation markers in urine pellet and tissue.

The approach employed for cfDNA study in BC diagnosis, follow-up, prognosis, and even to aid treatment decision making is also diverse.

#### 3.2.1. Analysis of Genomic Alterations

In BC, there are commonly altered oncogenes and tumor-suppressor genes, being fibroblast growth factor receptor 3 (*FGFR3*), phosphatidylinositol-4,5-bisphosphate 3-kinase, catalytic subunit alpha (*PIK3CA*), Erb-B2 receptor tyrosine kinase 2 (*ERBB2*), and epidermal growth factor receptor (*EGFR*) among the most studied [45]. Most studies have focused on the detection of mutations, copy number variation (CNVs), and insertions and deletions (INDELs) commonly found in solid tumor samples of BC patients, nicely reviewed by Sanli et al. [6] and detailed henceforth.

Russo et al. [46] compared the presence of the *TER*T 228 G>A/T mutation in cfDNA of urine and tumor specimens by droplet digital PCR (ddPCR). In those BC patients with the mutation present in tissue samples, the identification in urine cfDNA achieved a sensitivity of 92%. Hayashi et al. [47] analyzed *TERT* promoter and *FGFR3* hotspot mutations in urinary cfDNA (*TERT* 228C>T, *TERT* 250C>T, and *FGFR3* 249S>C) by ddPCR. In combination with cytology, these mutations achieved a sensitivity of 78.6% and a specificity of 96.0% in diagnosis and staging of upper tract urothelial carcinoma [47]. In another study, these authors [48] evidenced that the diagnostic sensitivity of this strategy outperformed that of the combination of cytology and UroVysion, an FDA-approved assay to aid in BC diagnosis.

Similarly, Christensen et al. [49] screened cfDNA for *FGFR3* and *PIK3CA* hotspot mutations and concluded that an increased presence of *FGFR3* and *PIK3CA* mutations in urine and plasma cfDNA are indicative of later progression and metastasis in BC.

Kim et al. [50] selected four candidate genes for BC detection from a tissue microarray: *CDC20, IQGAP3, TOP2A*, and *UBE2C*. Levels of *IQGAP3* in urine cfDNA seemed to best identify BC patients from healthy controls and patients with hematuria. Additionally, these authors found that the expression of *TOP2A* in urine cfDNA from BC patients was significantly higher than in healthy controls and patients with hematuria, and it was also higher in MIBC than in NMIBC patients [51].

Lee et al. [52] deep-sequenced nine genes frequently mutated in BC (*ARID1A*, *PIK3CA*, *FGFR3*, *HRAS*, *KMT2D*, *RB1*, *TP53*, *KDM6A*, and *STAG2*) to identify mutations, and performed shallow whole genome sequencing (sWGS) to detect CNVs. They evidenced that the genetic alterations identified were comparable between urinary DNA (both cfDNA and derived from exosomes) and tumor samples.

Following the idea that detection of mutations, copy number alterations (CNAs), and even novel fusion genes in BC may offer a great alternative for precision cancer treatment, Vandekerkhove et al. [53] tried to find genetic alterations in cfDNA by using a combination of whole exome sequencing and targeted sequencing. They analyzed a cohort of patients with aggressive BC and saw that the distribution of all alterations found was consistent with previous studies in localized MIBC. Furthermore, a novel *FGFR3* gene fusion was identified in consecutive samples from one patient.

In recent years, efforts have been made to develop specific gene panels to help diagnose BC out of cfDNA. Whole genome or exome sequencing seem to be the best options, since most existing alterations can be identified. However, it is also very expensive and sometimes the small amount of cfDNA isolated from liquid biopsies hamper this approach. Thus, specific gene panels are being developed to diagnose BC, evaluate the prognosis, the efficacy of a specific treatment, etc. Several gene panels are based on previously reported genomic alterations in the literature or in databases like the Catalogue of Somatic Mutations in Cancer (COSMIC) database, a global resource for information on somatic mutations in human cancer [54]. For instance, Christensen et al. [55] designed a 51-gene panel for deep targeted sequencing of plasma cfDNA by identifying frequently mutated genes in BC, validated by ddPCR.

Using the FDA-approved Guardant 360^®^ panel designed to sequence 73 genes in all solid cancers, Agarwal et al. [56] evidenced that the genomic profile of metastatic urothelial carcinoma was similar in cfDNA and tissue specimens. Additionally, they observed that the frequency of genomic alterations was similar in lower tract and upper tract metastatic urothelial carcinoma. Grivas et al. [57] evidenced that the genomic alterations in blood of advanced urothelial carcinoma patients were similar to those found in tissue, and also that alterations in *BRCA1* and *RAF1* have negative prognostic value, which may open the novel therapeutic approaches by targeting these alterations.

Ward et al. [41], by using a combination of publicly available data and in-house exome sequencing, designed a 23-gene panel which contains the most frequent somatic mutations. Those mutations were reliably detected in both urinary cfDNA and cellular DNA, and comparable to those present in tumor tissue.

Ou et al. [58] studied the mutational status of cfDNA from both urine and plasma using a 48-gene panel to help diagnose BC. They concluded that gene mutations in urine (both cfDNA and cellular DNA) had better concordance with cancer tissue than plasma cfDNA. As a consequence, they designed two gene panels, one for cfDNA with five genes and the other one for cellular DNA with seven genes, for BC diagnosis.

All the aforementioned studies evidence that the analysis of genomic alterations in cfDNA for BC diagnosis and prognosis might represent a clinical valuable tool once standardized that may reduce or even avoid the use of detrimental diagnostic techniques for the patients.

#### 3.2.2. Analysis of Gene Expression

An additional strategy explored to diagnose BC is the quantification of specific gene ratios in cfDNA. Brisuda et al. [59] analyzed the expression level of *GAPDH* in urine cfDNA and found a significant increase in BC patients compared to controls. Furthermore, Xu et al. [60] enhanced this approach by evaluating the ratio of expression of one up-regulated gene and seven downregulated genes. After validating their results in an independent cohort of patients, they concluded that the ratios *IQGAP3/BMP4* and *IQGAP3/FAM107A* were both significantly increased in urine cfDNA from BC patients compare to patients with hematuria. Next, they evidenced that the ratio *IQGAP3/BMP4* in urine cfDNA from NMIBC patients was associated with worse recurrence-free and progression-free survival [61].

As previously mentioned, ctDNA is a very small part of all cfDNA available, which may complicate ctDNA detection. In addition, cfDNA levels increase over time and in several clinical situations. Accordingly, Henriksen et al. [62] demonstrated that after any kind of trauma, cfDNA concentration increases in urine, but not ctDNA. This trauma-induced DNA can risk some postsurgical treatment decisions, so they monitored this trauma-induced changes in cfDNA concentration for up to 6 weeks after cancer surgery and measured both short (peak size: ~166 bp, probably non-ctDNA released from apoptosis) and long (smear size: > 1 kb, probably ctDNA released from necrosis). The levels of the short cfDNA fragment studied highly increased in week 1 and gradually decreased in weeks 2-4 before reaching preoperative level in week 5. In contrast, the levels of the long fragment studied hardly changed, being briefly higher in week 2 but similar to preoperative levels in all other weeks. They also performed this study in colorectal cancer and the levels of the short fragment were not as high as in BC. This would indicate that invasive procedures such as cystectomy may imply a severe trauma and thus may influence cfDNA levels [62].

Additionally, Vandekerkhove et al. [53] observed an increase in cfDNA yields but not in ctDNA levels in BC patients receiving chemotherapy, which highlights the importance of timing in the collection of liquid biopsies.

Cheng et al. [63] evidenced that MIBC patients had a higher proportion of long urine cfDNA fragments, as well as longer cfDNA fragments originating from ctDNA.

Finally, Casadio et al. [64,65] evaluated the integrity of urine cfDNA for early BC diagnosis. To that aim, they quantified long cfDNA fragments (>250 bp) of four oncogene regions known to increase its copy number in solid tumors in BC patients and healthy controls. The cfDNA integrity was reported to have high sensitivity (73%) and specificity (84%) in detecting BC in symptomatic patients.

In contrast to the study of genomic alterations, the analysis of gene expression in cfDNA for BC assessment still remains controversial. Disagreements arise among studies regarding the usefulness of assessing cfDNA or ctDNA, thus further studies are needed to clarify their applicability.

#### 3.2.3. Analysis of cfDNA Using Novel Technologies

In view of the fact that the amount of cfDNA found in liquid biopsies may be insufficient for multiplex genetic analyses, some efforts have been made in order to find a reliable method for the analysis of cfDNA. Soave et al. [66] evaluated whether multiplex ligation-dependent probe amplification (MLPA) was a good method to characterize CNVs in plasma and serum cfDNA. They tested different commercial kits for cfDNA isolation and checked whether MLPA worked in all of them. They concluded that MLPA was only suitable for cfDNA isolated from serum using the PME free-circulating DNA Extraction kit (PME kit, Analytik Jena, Jena, Germany).

Cheng et al. [63] studied methylation and CNAs after performing sWGS bisulfite sequencing and they were able to detect BC with a sensitivity of 93.5% (84.2% for low-grade NMIBC) and a specificity of 95.8%, and reflected stage and tumor size.

Ge et al. [67] developed a CNAs profile detector called UCdetector based on the results obtained with sWGS of urine cfDNA in different cancers, not only BC, but also kidney and prostate cancer. They also concluded that cfDNA analysis is more sensitive than that of urine cellular DNA.

Zhao et al. [68] developed a novel cell-free single-molecule unique primer extension resequencing (cf-SUPER) technology which detects cfDNA point mutations in urine samples to diagnose and stage BC. They selected 22 genes from the literature and the COSMIC database strongly associated with BC and designed the analysis of 740 hotspot mutations. They found that the cf-SUPER method can accurately detect mutations with allele frequencies as low as 0.01% and using as low as 1 ng DNA. The mutations were detected in urine cfDNA and tissue with a consistency of 82.76%, and the diagnostic consistency was 89.66%. Next, they evaluated the technology in a larger cohort of BC patients from the COSMIC database, and those mutations could be identified in more than 80% patients.

As mentioned, BC patients have a higher amount of cfDNA in blood and urine than healthy individuals. Detecting this increase over time by a simple method could represent a valuable diagnostic tool for BC. Accordingly, do Nascimento Alves et al. [69] compared the concentration of cfDNA isolated from blood and urine sediment that was collected during the course of BC treatment by Z-scan, a technique that can measure the nonlinear optical properties of liquid biopsies with more sensitivity than other optical techniques such as spectroscopy and fluorescence. Unlike spectrophotometry, Z-scan identified differences in cfDNA concentrations from blood and urine of BC patients and controls, as well as differences over time.

While more conventional techniques such as the analysis of genomic alterations or gene expression from urine cfDNA seem to gain importance in BC assessment, novel technologies are under development to overcome potential drawbacks of these techniques. Nonetheless, their applicability will depend on external validation studies and on the use of widely available equipment.

### 3.3. Analysis of cfDNA as Monitoring Biomarker for BC Treatment

ctDNA sequencing may also be used to monitor BC patients and seems to be a good option to detect treatment efficacy. Birkenkamp-Demtröder et al. [70] used three different methods to identify genomic variants in plasma, urine, and matching tumor tissue: WGS, whole exome sequencing, and mate-pair sequencing, and monitored the somatic variants by ddPCR. The analysis of genomic variants in ctDNA from the liquid biopsies was then used to create personalized assays for the patients to monitor their clinical outcomes. Even though they used a small cohort of patients, their study suggested that ctDNA can be detected in plasma and urine, even in patients with NMIBC, with high levels of ctDNA being detectable in patients with progressive disease, compared with low levels in samples from patients with recurrent disease especially in urine samples, which may indicate that ctDNA reflects how invasive the tumor may be rather than just its presence in the bladder and thus, ctDNA can be a useful tool for disease surveillance [70]. Two years later, the same group demonstrated early detection of metastatic relapse as well as indications of treatment response using plasma ctDNA analysis. When comparing ctDNA levels in patients before, during, and after treatment, a decrease was observed. They also observed that detection of ctDNA after cystectomy was associated with metastatic disease [71]. Following this study, Christensen et al. [72] concluded that ctDNA analysis can identify patients with metastatic relapse after cystectomy with a 100% sensitivity and 98% specificity, and its dynamics during chemotherapy was associated with disease recurrence. Furthermore, pathologic downstaging was associated with the presence of some mutations.

Cisplatin-based chemotherapy has been used for decades as therapy for metastatic urothelial carcinoma patients. Nonetheless, alternative therapies are under development for those who may not benefit from chemotherapy. Some studies have analyzed the role or checkpoint inhibitors, which block *PD-1* or its ligand *PD-L1*, although some patients cannot benefit from these therapies due to immune exclusion. Most of these patient’s tumors have mutations in *FGFR*3, so drug development for this target may be of great help [73]. Accordingly, Raja et al. [74] used Guardant 360^®^ to test whether alterations in cfDNA may predict survival in BC patients treated with an anti-*PD-L1* (Durvalumab). This emerging immunotherapy blocks this ligand´s response, thus preventing unregulated destructive inflammation [6]. They discovered that ctDNA variant allele frequencies reduction in time was related to a reduction in tumor volume, longer progression-free and overall survival, and may be a predictor of long-term benefit from immunotherapy in BC [74].

As previously mentioned, *FGFR3* is a good target for drug development. Infigratinib (BGJ398) is a potent and selective *FGFR1-3* inhibitor with significant activity in patients with advanced or metastatic urothelial carcinoma bearing *FGFR3* alterations. This treatment is currently in phase 2 of clinical trials. Bearing in mind that *FGFR3* is more frequently altered in upper tract urothelial carcinoma compared to urothelial carcinoma of the bladder [75], Pal et al. [73,76] evaluated the pattern of response to therapy of patients with these tumor types. As expected, they evidenced a greater proportion of genomic alterations in patients with upper tract urothelial carcinoma, including higher frequencies of *FGFR3*-*TACC*3 fusions and *FGFR3* 248R>C mutations and a lower frequency of *FGFR3* 249S>C mutations, and also a greater response to treatment in these patients. This fact supported the start of a phase 3 trial for Infigratinib [73,76].

In summary, although additional studies are needed to validate the abovementioned approaches, the assessment of urine ctDNA in BC patients to monitor BC treatment may become a suitable method in the near future.

## 4. Conclusions

The analysis of liquid biopsies has recently arisen as a magnificent option for disease diagnosis, follow-up, prognosis, and even for treatment decision-making in clinical practice benefitting from its low invasiveness. In the particular scenario of BC, cfDNA represents one of the most informative biomarkers. Its applications have been thoroughly reviewed herein.

In overview, the majority of the studies regarding cfDNA and BC have focused on comparing genomic alterations (somatic mutations, CNVs, INDELs, gene fusions, degree of methylation, etc.) found in tissue from BC patients in the past with those found in liquid biopsies. Presently, there is an urgent need for proving beyond a doubt that liquid biopsies represent a good substitute for tissue biopsies. To that aim tissue biomarkers have been explored in urine cfDNA and cellular DNA and in blood, either by sequencing the whole genome, exomes, using panels exploring specific candidate genes, analyzing gene expression, etc. All in all, the final goal pursued is to clearly distinguish BC patients from healthy controls, and even stage BC patients. An additional future goal of cfDNA analysis in liquid biopsies is to monitor and tailor BC therapies and also to evaluate resistance to treatments.

Nevertheless, additional efforts are needed to establish a standardised method to sample collection, preprocessing liquid biopsies, cfDNA isolation from different liquid biopsies, and the selection of the most informative technique for its study. This clearly evidences that additional research in this field is needed before implementing the analysis of cfDNA from liquid biopsies in BC management as either complementing current diagnostic methods or even substituting them in the near future.

## Data Availability

Any data reviewed in this manuscript should be consulted in the original articles cited.

## Supplementary Materials

The following are available online at https://www.mdpi.com/2072-6694/13/6/1448/s1, Table S1: Description of the studies regarding cfDNA in liquid biopsies as potential biomarker for bladder cancer.

## References

[1] Bray F., Ferlay J., Soerjomataram I., Siegel R.L., Torre L.A., Jemal A. (2018). Global cancer statistics 2018: GLOBOCAN estimates of incidence and mortality worldwide for 36 cancers in 185 countries. CA Cancer J. Clin..

[2] Siegel R.L., Miller K.D., Jemal A. (2019). Cancer statistics, 2019. CA Cancer J. Clin..

[3] Svatek R.S., Hollenbeck B.K., Holmäng S., Lee R., Kim S.P., Stenzl A., Lotan Y. (2014). The economics of bladder cancer: Costs and considerations of caring for this disease. Eur. Urol..

[4] Cumberbatch M.G.K., Noon A.P. (2019). Epidemiology, aetiology and screening of bladder cancer. Transl. Androl. Urol..

[5] MacmillanCancerSupport Staging and Grading of Bladder Cancer. https://www.macmillan.org.uk/information-and-support/bladder-cancer/non-invasive-bladder-cancer/treating/treatment-decisions/understanding-your-diagnosis/staging-grading.html.

[6] Sanli O., Dobruch J., Knowles M.A., Burger M., Alemozaffar M., Nielsen M.E., Lotan Y. (2017). Bladder cancer. Nat. Rev. Dis. Primers.

[7] Kirkali Z., Chan T., Manoharan M., Algaba F., Busch C., Cheng L., Kiemeney L., Kriegmair M., Montironi R., Murphy W.M. (2005). Bladder cancer: Epidemiology, staging and grading, and diagnosis. Urology.

[8] Kamoun A., de Reyniès A., Allory Y., Sjödahl G., Robertson A.G., Seiler R., Hoadley K.A., Groeneveld C.S., Al-Ahmadie H., Choi W. (2020). A Consensus Molecular Classification of Muscle-invasive Bladder Cancer. Eur. Urol..

[9] MacVicar A.D. (2000). Bladder cancer staging. BJU Int..

[10] (2002). PDQ Cancer Information Summaries [Internet]: Bladder Cancer Treatment (PDQ®).

[11] Zhu C.Z., Ting H.N., Ng K.H., Ong T.A. (2019). A review on the accuracy of bladder cancer detection methods. J. Cancer.

[12] Wu S., Zheng J., Li Y., Wu Z., Shi S., Huang M., Yu H., Dong W., Huang J., Lin T. (2018). Development and Validation of an MRI-Based Radiomics Signature for the Preoperative Prediction of Lymph Node Metastasis in Bladder Cancer. EBioMedicine.

[13] Yafi F.A., Brimo F., Steinberg J., Aprikian A.G., Tanguay S., Kassouf W. (2015). Prospective analysis of sensitivity and specificity of urinary cytology and other urinary biomarkers for bladder cancer. Urol. Oncol..

[14] Lee H.-H., Kim S.H. (2020). Review of non-invasive urinary biomarkers in bladder cancer. Transl. Cancer Res..

[15] Fernández-Lázaro D., Hernández J.L.G., García A.C., Castillo A.C.D., Hueso M.V., Cruz-Hernández J.J. (2020). Clinical Perspective and Translational Oncology of Liquid Biopsy. Diagnostics.

[16] Poulet G., Massias J., Taly V. (2019). Liquid Biopsy: General Concepts. Acta Cytol..

[17] Fernández-Lázaro D., García Hernández J.L., García A.C., Córdova Martínez A., Mielgo-Ayuso J., Cruz-Hernández J.J. (2020). Liquid Biopsy as Novel Tool in Precision Medicine: Origins, Properties, Identification and Clinical Perspective of Cancer’s Biomarkers. Diagnostics.

[18] Riethdorf S., Soave A., Rink M. (2017). The current status and clinical value of circulating tumor cells and circulating cell-free tumor DNA in bladder cancer. Transl. Androl. Urol..

[19] Shu Y., Wu X., Tong X., Wang X., Chang Z., Mao Y., Chen X., Sun J., Wang Z., Hong Z. (2017). Circulating Tumor DNA Mutation Profiling by Targeted Next Generation Sequencing Provides Guidance for Personalized Treatments in Multiple Cancer Types. Sci. Rep..

[20] Corcoran R.B., Chabner B.A. (2018). Application of Cell-free DNA Analysis to Cancer Treatment. N. Engl. J. Med..

[21] Huang C.C., Du M., Wang L. (2019). Bioinformatics Analysis for Circulating Cell-Free DNA in Cancer. Cancers.

[22] Huang J., Wang L. (2019). Cell-Free DNA Methylation Profiling Analysis-Technologies and Bioinformatics. Cancers.

[23] Bronkhorst A.J., Ungerer V., Holdenrieder S. (2019). The emerging role of cell-free DNA as a molecular marker for cancer management. Biomol. Detect. Quantif..

[24] Smith C.G., Moser T., Mouliere F., Field-Rayner J., Eldridge M., Riediger A.L., Chandrananda D., Heider K., Wan J.C.M., Warren A.Y. (2020). Comprehensive characterization of cell-free tumor DNA in plasma and urine of patients with renal tumors. Genome Med..

[25] Diaz L.A., Bardelli A. (2014). Liquid biopsies: Genotyping circulating tumor DNA. J. Clin. Oncol..

[26] Wan J.C.M., Massie C., Garcia-Corbacho J., Mouliere F., Brenton J.D., Caldas C., Pacey S., Baird R., Rosenfeld N. (2017). Liquid biopsies come of age: Towards implementation of circulating tumour DNA. Nat. Rev. Cancer.

[27] Mouliere F., Chandrananda D., Piskorz A.M., Moore E.K., Morris J., Ahlborn L.B., Mair R., Goranova T., Marass F., Heider K. (2018). Enhanced detection of circulating tumor DNA by fragment size analysis. Sci. Transl. Med..

[28] Crisafulli G., Mussolin B., Cassingena A., Montone M., Bartolini A., Barault L., Martinetti A., Morano F., Pietrantonio F., Sartore-Bianchi A. (2019). Whole exome sequencing analysis of urine trans-renal tumour DNA in metastatic colorectal cancer patients. ESMO Open.

[29] Nakano K., Yamamoto Y., Yamamichi G., Yumiba S., Tomiyama E., Matsushita M., Koh Y., Hayashi Y., Wang C., Ishizuya Y. (2020). Fragmentation of cell-free DNA is induced by upper-tract urothelial carcinoma-associated systemic inflammation. Cancer Sci..

[30] Agostini M., Enzo M.V., Bedin C., Belardinelli V., Goldin E., Del Bianco P., Maschietto E., D’Angelo E., Izzi L., Saccani A. (2012). Circulating cell-free DNA: A promising marker of regional lymphonode metastasis in breast cancer patients. Cancer Biomark.

[31] Fawzy A., Sweify K.M., El-Fayoumy H.M., Nofal N. (2016). Quantitative analysis of plasma cell-free DNA and its DNA integrity in patients with metastatic prostate cancer using ALU sequence. J. Egypt Natl. Cancer Inst..

[32] Lu T., Li J. (2017). Clinical applications of urinary cell-free DNA in cancer: Current insights and promising future. Am. J. Cancer Res..

[33] Umansky S.R., Tomei L.D. (2006). Transrenal DNA testing: Progress and perspectives. Expert Rev. Mol. Diagn..

[34] García Moreira V., Prieto García B., de la Cera Martínez T., Alvarez Menéndez F.V. (2009). Elevated transrenal DNA (cell-free urine DNA) in patients with urinary tract infection compared to healthy controls. Clin. Biochem..

[35] Wang X., Meng Q., Wang C., Li F., Zhu Z., Liu S., Shi Y., Huang J., Chen S., Li C. (2017). Investigation of transrenal KRAS mutation in late stage NSCLC patients correlates to disease progression. Biomarkers.

[36] Lanman R.B., Mortimer S.A., Zill O.A., Sebisanovic D., Lopez R., Blau S., Collisson E.A., Divers S.G., Hoon D.S., Kopetz E.S. (2015). Analytical and Clinical Validation of a Digital Sequencing Panel for Quantitative, Highly Accurate Evaluation of Cell-Free Circulating Tumor DNA. PLoS ONE.

[37] Li P., Ning J., Luo X., Du H., Zhang Q., Zhou G., Du Q., Ou Z., Wang L., Wang Y. (2019). New method to preserve the original proportion and integrity of urinary cell-free DNA. J. Clin. Lab. Anal..

[38] Martignano F. (2019). Cell-Free DNA: An Overview of Sample Types and Isolation Procedures. Methods Mol. Biol..

[39] Augustus E., Van Casteren K., Sorber L., van Dam P., Roeyen G., Peeters M., Vorsters A., Wouters A., Raskin J., Rolfo C. (2020). The art of obtaining a high yield of cell-free DNA from urine. PLoS ONE.

[40] Emaus M.N., Varona M., Anderson J.L. (2019). Sequence-specific preconcentration of a mutation prone KRAS fragment from plasma using ion-tagged oligonucleotides coupled to qPCR compatible magnetic ionic liquid solvents. Anal. Chim. Acta.

[41] Ward D.G., Gordon N.S., Boucher R.H., Pirrie S.J., Baxter L., Ott S., Silcock L., Whalley C.M., Stockton J.D., Beggs A.D. (2019). Targeted deep sequencing of urothelial bladder cancers and associated urinary DNA: A 23-gene panel with utility for non-invasive diagnosis and risk stratification. BJU Int..

[42] Togneri F.S., Ward D.G., Foster J.M., Devall A.J., Wojtowicz P., Alyas S., Vasques F.R., Oumie A., James N.D., Cheng K.K. (2016). Genomic complexity of urothelial bladder cancer revealed in urinary cfDNA. Eur. J. Hum. Genet..

[43] Stasik S., Salomo K., Heberling U., Froehner M., Sommer U., Baretton G.B., Ehninger G., Wirth M.P., Thiede C., Fuessel S. (2019). Evaluation of TERT promoter mutations in urinary cell-free DNA and sediment DNA for detection of bladder cancer. Clin. Biochem..

[44] Hentschel A.E., Nieuwenhuijzen J.A., Bosschieter J., Splunter A.P.V., Lissenberg-Witte B.I., Voorn J.P.V., Segerink L.I., Moorselaar R., Steenbergen R.D.M. (2020). Comparative Analysis of Urine Fractions for Optimal Bladder Cancer Detection Using DNA Methylation Markers. Cancers.

[45] Millis S.Z., Bryant D., Basu G., Bender R., Vranic S., Gatalica Z., Vogelzang N.J. (2015). Molecular profiling of infiltrating urothelial carcinoma of bladder and nonbladder origin. Clin. Genitourin Cancer.

[46] Russo I.J., Ju Y., Gordon N.S., Zeegers M.P., Cheng K.K., James N.D., Bryan R.T., Ward D.G. (2018). Toward Personalised Liquid Biopsies for Urothelial Carcinoma: Characterisation of ddPCR and Urinary cfDNA for the Detection of the TERT 228G>A/T Mutation. Bladder Cancer.

[47] Hayashi Y., Fujita K., Matsuzaki K., Matsushita M., Kawamura N., Koh Y., Nakano K., Wang C., Ishizuya Y., Yamamoto Y. (2019). Diagnostic potential of TERT promoter and FGFR3 mutations in urinary cell-free DNA in upper tract urothelial carcinoma. Cancer Sci..

[48] Hayashi Y., Fujita K., Matsuzaki K., Eich M.L., Tomiyama E., Matsushita M., Koh Y., Nakano K., Wang C., Ishizuya Y. (2020). Clinical Significance of Hotspot Mutation Analysis of Urinary Cell-Free DNA in Urothelial Bladder Cancer. Front. Oncol..

[49] Christensen E., Birkenkamp-Demtröder K., Nordentoft I., Høyer S., van der Keur K., van Kessel K., Zwarthoff E., Agerbæk M., Ørntoft T.F., Jensen J.B. (2017). Liquid Biopsy Analysis of FGFR3 and PIK3CA Hotspot Mutations for Disease Surveillance in Bladder Cancer. Eur. Urol..

[50] Kim W.T., Kim Y.H., Jeong P., Seo S.P., Kang H.W., Kim Y.J., Yun S.J., Lee S.C., Moon S.K., Choi Y.H. (2018). Urinary cell-free nucleic acid IQGAP3: A new non-invasive diagnostic marker for bladder cancer. Oncotarget.

[51] Kim Y.H., Yan C., Lee I.S., Piao X.M., Byun Y.J., Jeong P., Kim W.T., Yun S.J., Kim W.J. (2016). Value of urinary topoisomerase-IIA cell-free DNA for diagnosis of bladder cancer. Investig. Clin. Urol..

[52] Lee D.H., Yoon H., Park S., Kim J.S., Ahn Y.H., Kwon K., Lee D., Kim K.H. (2018). Urinary Exosomal and cell-free DNA Detects Somatic Mutation and Copy Number Alteration in Urothelial Carcinoma of Bladder. Sci. Rep..

[53] Vandekerkhove G., Todenhöfer T., Annala M., Struss W.J., Wong A., Beja K., Ritch E., Brahmbhatt S., Volik S.V., Hennenlotter J. (2017). Circulating Tumor DNA Reveals Clinically Actionable Somatic Genome of Metastatic Bladder Cancer. Clin. Cancer Res..

[54] Forbes S.A., Bhamra G., Bamford S., Dawson E., Kok C., Clements J., Menzies A., Teague J.W., Futreal P.A., Stratton M.R. (2008). The Catalogue of Somatic Mutations in Cancer (COSMIC). Curr. Protoc. Hum. Genet..

[55] Christensen E., Nordentoft I., Vang S., Birkenkamp-Demtröder K., Jensen J.B., Agerbæk M., Pedersen J.S., Dyrskjøt L. (2018). Optimized targeted sequencing of cell-free plasma DNA from bladder cancer patients. Sci. Rep..

[56] Agarwal N., Pal S.K., Hahn A.W., Nussenzveig R.H., Pond G.R., Gupta S.V., Wang J., Bilen M.A., Naik G., Ghatalia P. (2018). Characterization of metastatic urothelial carcinoma via comprehensive genomic profiling of circulating tumor DNA. Cancer.

[57] Grivas P., Lalani A.A., Pond G.R., Nagy R.J., Faltas B., Agarwal N., Gupta S.V., Drakaki A., Vaishampayan U.N., Wang J. (2020). Circulating Tumor DNA Alterations in Advanced Urothelial Carcinoma and Association with Clinical Outcomes: A Pilot Study. Eur. Urol. Oncol..

[58] Ou Z., Li K., Yang T., Dai Y., Chandra M., Ning J., Wang Y., Xu R., Gao T., Xie Y. (2020). Detection of bladder cancer using urinary cell-free DNA and cellular DNA. Clin. Transl. Med..

[59] Brisuda A., Pazourkova E., Soukup V., Horinek A., Hrbáček J., Capoun O., Svobodova I., Pospisilova S., Korabecna M., Mares J. (2016). Urinary Cell-Free DNA Quantification as Non-Invasive Biomarker in Patients with Bladder Cancer. Urol. Int..

[60] Xu Y., Kim Y.H., Jeong P., Piao X.M., Byun Y.J., Kang H.W., Kim W.T., Lee J.Y., Kim I.Y., Moon S.K. (2019). Diagnostic value of combined IQGAP3/BMP4 and IQGAP3/FAM107A expression ratios in urinary cell-free DNA for discriminating bladder cancer from hematuria. Urol. Oncol..

[61] Xu Y., Kim Y.H., Jeong P., Piao X.M., Byun Y.J., Seo S.P., Kang H.W., Kim W.T., Lee J.Y., Ryu D.H. (2019). Urinary Cell-Free DNA IQGAP3/BMP4 Ratio as a Prognostic Marker for Non-Muscle-Invasive Bladder Cancer. Clin. Genitourin Cancer.

[62] Henriksen T.V., Reinert T., Christensen E., Sethi H., Birkenkamp-Demtröder K., Gögenur M., Gögenur I., Zimmermann B.G., Dyrskjøt L., Andersen C.L. (2020). The effect of surgical trauma on circulating free DNA levels in cancer patients-implications for studies of circulating tumor DNA. Mol. Oncol..

[63] Cheng T.H.T., Jiang P., Teoh J.Y.C., Heung M.M.S., Tam J.C.W., Sun X., Lee W.S., Ni M., Chan R.C.K., Ng C.F. (2019). Noninvasive Detection of Bladder Cancer by Shallow-Depth Genome-Wide Bisulfite Sequencing of Urinary Cell-Free DNA for Methylation and Copy Number Profiling. Clin. Chem..

[64] Casadio V., Calistri D., Tebaldi M., Bravaccini S., Gunelli R., Martorana G., Bertaccini A., Serra L., Scarpi E., Amadori D. (2013). Urine cell-free DNA integrity as a marker for early bladder cancer diagnosis: Preliminary data. Urol. Oncol..

[65] Casadio V., Salvi S., Martignano F., Gunelli R., Ravaioli S., Calistri D. (2017). Cell-Free DNA Integrity Analysis in Urine Samples. J. Vis. Exp..

[66] Soave A., Chun F.K., Hillebrand T., Rink M., Weisbach L., Steinbach B., Fisch M., Pantel K., Schwarzenbach H. (2017). Copy number variations of circulating, cell-free DNA in urothelial carcinoma of the bladder patients treated with radical cystectomy: A prospective study. Oncotarget.

[67] Ge G., Peng D., Guan B., Zhou Y., Gong Y., Shi Y., Hao X., Xu Z., Qi J., Lu H. (2020). Urothelial Carcinoma Detection Based on Copy Number Profiles of Urinary Cell-Free DNA by Shallow Whole-Genome Sequencing. Clin. Chem..

[68] Zhao C., Pan Y., Wang Y., Li Y., Han W., Lu L., Tang W., Li P., Ou Z., Zhang M. (2020). A novel cell-free single-molecule unique primer extension resequencing (cf-SUPER) technology for bladder cancer non-invasive detection in urine. Transl. Androl. Urol..

[69] Do Nascimento Alves S., Lavalhegas Hallack M., Moreira Perez M., da Costa Aguiar Alves B., da Silva L.H., Afonso Fonseca F.L. (2019). Application of the Z-scan technique for the detection of CFCDNA (cell-free circulating DNA) and urine DNA (uDNA) in patients with bladder cancer. Photodiagnosis Photodyn. Ther..

[70] Birkenkamp-Demtröder K., Nordentoft I., Christensen E., Høyer S., Reinert T., Vang S., Borre M., Agerbæk M., Jensen J.B., Ørntoft T.F. (2016). Genomic Alterations in Liquid Biopsies from Patients with Bladder Cancer. Eur. Urol..

[71] Birkenkamp-Demtröder K., Christensen E., Nordentoft I., Knudsen M., Taber A., Høyer S., Lamy P., Agerbæk M., Jensen J.B., Dyrskjøt L. (2018). Monitoring Treatment Response and Metastatic Relapse in Advanced Bladder Cancer by Liquid Biopsy Analysis. Eur. Urol..

[72] Christensen E., Birkenkamp-Demtröder K., Sethi H., Shchegrova S., Salari R., Nordentoft I., Wu H.T., Knudsen M., Lamy P., Lindskrog S.V. (2019). Early Detection of Metastatic Relapse and Monitoring of Therapeutic Efficacy by Ultra-Deep Sequencing of Plasma Cell-Free DNA in Patients With Urothelial Bladder Carcinoma. J. Clin. Oncol..

[73] Pal S.K., Bajorin D., Dizman N., Hoffman-Censits J., Quinn D.I., Petrylak D.P., Galsky M.D., Vaishampayan U., De Giorgi U., Gupta S. (2020). Infigratinib in upper tract urothelial carcinoma versus urothelial carcinoma of the bladder and its association with comprehensive genomic profiling and/or cell-free DNA results. Cancer.

[74] Raja R., Kuziora M., Brohawn P.Z., Higgs B.W., Gupta A., Dennis P.A., Ranade K. (2018). Early Reduction in ctDNA Predicts Survival in Patients with Lung and Bladder Cancer Treated with Durvalumab. Clin. Cancer Res..

[75] Audenet F., Isharwal S., Cha E.K., Donoghue M.T.A., Drill E.N., Ostrovnaya I., Pietzak E.J., Sfakianos J.P., Bagrodia A., Murugan P. (2019). Clonal Relatedness and Mutational Differences between Upper Tract and Bladder Urothelial Carcinoma. Clin. Cancer Res..

[76] Pal S.K., Rosenberg J.E., Hoffman-Censits J.H., Berger R., Quinn D.I., Galsky M.D., Wolf J., Dittrich C., Keam B., Delord J.P. (2018). Efficacy of BGJ398, a Fibroblast Growth Factor Receptor 1-3 Inhibitor, in Patients with Previously Treated Advanced Urothelial Carcinoma with FGFR3 Alterations. Cancer Discov..

