# Supplementary material for: Circulating Cell-Free DNA in Liquid Biopsies as Potential Biomarker for Bladder Cancer: A Systematic Review
Source: Cancers (Basel). 2021 Mar 22;13(6):1448. doi: 10.3390/cancers13061448 (PMC8005001; doi:10.3390/cancers13061448)
Supplement: Supplementary file 1 [file cancers-13-01448-s001.pdf]

Supplementary Material: Circulating Cell-Free DNA in Liquid Biopsies as Potential Biomarker for Bladder Cancer: A Systematic Review

Raquel Herranz, Julia Oto, Emma Plana, Álvaro Fernández-Pardo, Fernando Cana, Manuel Martínez-Sarmiento, César D. Vera-Donoso, Francisco España and Pilar Medina

Table S1. Description of the studies regarding cfDNA in liquid biopsies as potential biomarker for bladder cancer.

Reference	N	Liquid Biopsy	Sample Volume and cfDNA Isolation Kit Used	Detection Method	Genes Studied[1]	Clinically Relevant Findings
Zhao et al. 2020 [1]	47 BC patients<br>53 controls	Urine	Unspecified volume of urine supernatant<br>Magnetic Serum/Plasma DNA Maxi Kit (Tiangen Biotechnology, China)	A cell-free single-molecule unique primer extension resequencing (cf-SUPER) technology	22-gene targeted sequencing panel (uriprier panel)	Development of a novel cf-SUPER technology which detects cfDNA point mutations of cfDNA with diagnostic and staging purposes
Hayashi et al. 2020 [2]	Cohort 1:<br>74 BC patients<br>52 controls (benign hematuria)<br>Cohort 2:<br>40 BC patients, 36 patients under surveillance after surgery	Urine	12 mL urine supernatant<br>QIAamp Circulating Nucleic Acid Kit (Qiagen, Hilden, Germany)	ddPCR	*TERT* promoter and *FGFR3*	*TERT* promoter and *FGFR3* hotspot mutation analysis in urinary cfDNA by ddPCR combined with cytology has a higher sensitivity than when combined with UroVysion
Henriksen et al. 2020 [3]	47 MIBC patients	Plasma	8 mL plasma<br>QIAamp Circulating Nucleic Acids Kit (Qiagen)<br>Using dual-sided selection with SPRI beads, cfDNA was separated in two fractions with short (< 1 kb) and long (> 1 kb) DNA fragments, respectively	The size-separated cfDNA was profiled on a LabChip GX (PerkinElmer, Waltham, MA, USA) and quantified using the high-sensitivity QuantiT dsDNA assay (Invitrogen, Carlsbad, CA, USA)	N/A	After any kind of trauma, cfDNA concentration increases, but not ctDNA, which remains similar to pre-operative levels

Reference	N	Liquid Biopsy	Sample Volume and cfDNA Isolation Kit Used	Detection Method	Genes Studied[1]	Clinically Relevant Findings
Hentschel et al. 2020 [4]	14 urothelial BC patients	Urine	15 mL urine supernatant QuickDNA™ Urine Kit (Zymo Research, Orange, CA, USA)	DNA methylation analysis after bisulfite conversion by multiplex qMSP	*FAM19A4*, *GHSR*, *MAL*, *PHACTR3*, *PRDM14*, *SST* and *ZIC1*	Correlation for most methylation markers studied between urine cfDNA and cellular DNA and tissue specimen, being greater in cellular DNA
Pal et al. 2020 [5]	67 BC patients with oral Infigratinib	Plasma	Unspecified volume of plasma QIAamp Circulating Nucleic Acid Kit (Qiagen)	WGS libraries generated with the Illumina TruSeq Nano DNA Library Prep Kit (Illumina, San Diego, California) 600-gene pan-cancer panel on an Illumina HiSeq 2500 System (Illumina)	600-gene pan-cancer panel	Differences in the cumulative genomic profile observed between patients with upper-tract urothelial carcinoma and BC Phase-2 clinical trial of Infigratinib (BGJ398)
Ou et al. 2020 [6]	92 BC patients 33 controls	Urine Plasma	Unspecified volume of urine supernatant and plasma GenMag Circulating Nucleic Acid Kit (GenMag Biotechnology, Beijing, China)	NGS-based cfDNA allelic molecule-counting system termed the cfDNA barcode-enabled single-molecule test (cfBEST)	48-genes panel	Identification of a 5-genes panel for urine cfDNA (*TERT*, *FGFR3*, *TP53*, *PIK3CA*, and *KRAS*) to identify BC patients from hematuria patients
Ge et al. 2020 [7]	Original cohort: 65 UC patients 95 controls Validation cohort: CNA data from 410 UCBs from The Cancer Genome Atlas	Urine	Unspecified volume of urine supernatant ZYMO Quick-DNA Urine Kit (D3061, Zymo Research) cfDNA was size selected with 0.6 AMPure XP beads (Beckman Coulter) to remove large DNA fragments, and the remaining supernatant was purified by 0.3 beads to enrich the fragments with a range of approximately 100 to 300 base pairs (bp) to create libraries, which were sequenced on a HiSeq 10 system	CNAs screening profile by sWGS UCdetector to identify CNAs	N/A	Development of UCdetector based on a CNAs profile after screening using sWGS

Reference	N	Liquid Biopsy	Sample Volume and cfDNA Isolation Kit Used	Detection Method	Genes Studied[1]	Clinically Relevant Findings
Grivas et al. 2020 [8]	124 advanced UC patients	Blood	Unspecified volume, sent to Guardant Health (Redwood City, CA, USA) for cfDNA analysis (Guardant360)	unknown	73-gene sequencing panel (Guardant360)	Genomic alterations detected in most advanced urothelial carcinoma patients, results comparable with tissue<br>Alterations in *BRCA1* and *RAF1* have negative prognostic value
Xu et al. 2019 [9]	103 NMIBC patients	Urine	1 mL urine supernatant<br>QIAquick gel extraction kit (Qiagen)	qPCR	*IQGAP3*/*BMP4* and *IQGAP3*/*FAM107A* ratios	High *IQGAP3*/*BMP4* ratio in urine cfDNA was associated with worse recurrence-free and progression-free survival
Ward et al. 2019 [10]	261 BC patients with SMs	Urine	Unspecified volume of urine supernatant<br>Quick-DNA Urine kits (Zymo Research)	Sequencing performed by a 23-genes panel	A 23-genes panel	Development of a 23-genes panel which contains the most frequent SMs in BCs patients<br>Those SMs were reliably detected in both urine cfDNA and cellular DNA and comparable to those present in tumour tissue
Emaus et al. 2019 [11]	Commercially available lyophilized plasma	Plasma	Unspecified volume of plasma<br>ITO-MIL	DNA quantified by adding the DNA-enriched MIL to the qPCR buffer to streamline the extraction procedure	*KRAS*	Design of the ITO-MIL method, a selective method with the ability to rapidly preconcentrate target DNA from diluted plasma, to isolate ctDNA from clinical samples
Christensen et al. 2019 [12]	68 patients with locally advanced BC	Plasma	A median of 7.5 mL of plasma (range: 1-10 mL)<br>QIAamp Circulating Nucleic Acid kit (Qiagen)	Plasma multiplex-PCR NGS<br>Sequencing performed in an Illumina HiSeq 2500 (Illumina)<br>Missense mutations analyzed using PolyPhen2 and MutationAssessor	34 DNA damage response (DDR)–associated genes	ctDNA analysis can identify patients with metastatic relapse after cystectomy with a 100% sensitivity and 98% specificity, and its dynamics during chemotherapy were associated with disease recurrence<br>Pathologic downstaging was associated with some mutations

**Reference**	**N**	**Liquid Biopsy**	**Sample Volume and cfDNA Isolation Kit Used**	**Detection Method**	**Genes Studied**[1]	**Clinically Relevant Findings**
Cheng et al. 2019 [13]	46 BC patients<br>39 controls	Urine	10 mL urine supernatant<br>Samples were supplemented with 15 mL 6 mol/L guanidine thiocyanate (Sigma–Aldrich) and 1 mL resin (Wizard Plus Minipreps DNA Purification System; Promega, Madison, WI, USA)<br>The resin-DNA complex was isolated, washed and eluted using the Wizard Plus Minipreps DNA Purification System	qPCR<br>sWGS-bisulfite sequencing of urinary cfDNA	N/A	A combination of methylome and CNAs were able to detect BC with a sensitivity of 93.5% (84.2% for low-grade non-muscle invasive BC) and a specificity of 95.8%, and reflected stage and tumour size<br>MIBC was associated with a higher proportion of long cfDNA, as well as longer ctDNA fragments
Hayashi et al. 2019 [14]	153 BC patients, including 56 upper-tract urothelial carcinoma patients	Urine	A median of 12 mL urine supernatant (range 4-32 mL)<br>QIAamp Circulating Nucleic Acid Kit (Qiagen)	ddPCR	*TERT* promoter and *FGFR3*	Analysis of *TERT* promoter and *FGFR3* hotspot mutations in cfDNA is sensible enough to be used in clinical practice and, in combination with cytology, could be used to diagnose and stage upper-tract urothelial carcinoma
do Nascimento Alves et al. 2019 [15]	30 BC patients	Plasma	1 mL plasma<br>GFXTM kit (Amersham Pharmacia Biotech, Inc, Piscataway, NJ, USA)	Z-scan and spectrophotometry	N/A	Unlike spectrophotometry, z-identified differences in cfDNA concentrations from blood and urine of BC patients and controls, as well as a differences over time
Stasik et al. 2019 [16]	53 BC patients<br>36 controls	Urine	Unspecified volume of urine supernatant<br>QIAamp viral RNA Mini kit (Qiagen)	qPCR and sequencing	*TERT* promoter	cfDNA seems to provide no advantage in diagnostic potential compared to DNA found in urine sediment when analysing two abundant point-mutations (228C>T/250C>T) in the *TERT* promoter

Reference	N	Liquid Biopsy	Sample Volume and cfDNA Isolation Kit Used	Detection Method	Genes Studied¹	Clinically Relevant Findings
Xu et al. 2019 [17]	Screening cohort:<br>40 BC patients<br>41 controls<br>Validation cohort:<br>149 BC patients<br>125 controls	Urine	1 mL urine supernatant<br>QIAquick gel extraction kit (Qiagen)	qPCR	Seven candidate genes-ratios	The ratios IQGAP3/BMP4 and IQGAP3/*FAM107A* in cfDNA were significantly increased in BC patients compared with those with hematuria, so they could be used as non-invasive urine-based diagnostic markers
Lee et al. 2018 [18]	9 BC patients	Urine	2–4 mL urine supernatant<br>MagMax Cell-Free DNA Isolation Kit (Thermo Fisher Scientific, Waltham, MA, USA)	Target gene captured with customized probes and a Celemics target capture kit<br>NGS performed withthe Illumina NextSeq. 500 platform (Illumina)<br>sWGS was used for the detection of CNV	*ARID1A*, *PIK3CA*, *FGFR3*, *HRAS*, *KMT2D*, *RB1*, *TP53*, *KDM6A* and *STAG*	The genetic alterations found in cfDNA and derived from exosomes are comparable those found in tumour samples
Li et al. 2019 [19]	24 BC patients	Urine	1.8 mL urine supernatant<br>MagMAX™ Cell-Free DNA Isolation kit (Applied Biosystems Inc., Foster City, CA, USA)	qPCR<br>cfDNA fragments distribution was analyzed by Agilent 2200, and the frequency of specific mutations of urinary system disease was detected by NGS method	*ACTB* (*ACTB-41* and *ACB-127*)	Development of standardised urine collection tubes to prevent cfDNA degradation and maintain urine cells in their original form during the sample collection process, ensuring stabilization of the original proportion and integrity of urine cfDNA, and minimising the background noise caused by urinary cellular DNA releasing
Raja et al. 2018 [20]	29 UC patients	Plasma	1 mL plasma<br>QIAamp Circulating Nucleic Acid Kit (Qiagen)<br>Concentrated using Agencourt Ampure XP beads (Beckman Coulter, Brea, CA, USA)	NGS-based 73-genes panel (Guardant360)	NGS-based 73-genes panel (Guardant360)	ctDNA VAF reduction in time (after Durvalumab treatment) seems to be related to reduction of tumour volume, longer progression-free and overall survival, and may be a predictor of long-term benefit from immunotherapy in BC patients

Reference	N	Liquid Biopsy	Sample Volume and cfDNA Isolation Kit Used	Detection Method	Genes Studied[1]	Clinically Relevant Findings
Kim et al. 2018 [21]	92 BC patients<br>120 healthy controls with hematuria	Urine	1 mL urine supernatant<br>QIAamp Circulating Nucleic Acid Kit (Qiagen)	RT-qPCR	*CDC20, IQGAP3, TOP2A, UBE2C*	Levels of *IQGAP3* in urine cfDNA of BC patients were significantly higher than those in healthy controls or patients with hematuria
Agarwal et al. 2018 [22]	369 metastatic UC patients (294 mLTUC and 75 mUTUC)	Blood	1.5-5 mL plasma<br>Concentrated and size-selected using Agencourt Ampure XP beads (Beckman Coulter) [23]	Guardant360 platform	NGS-based 73-gene panel (Guardant360)	The genomic profile of metastatic urothelial carcinoma was similar in cfDNA and tissue specimens<br>The frequency of genomic alterations is similar between mLTUC and mUTUC patients
Russo et al. 2018 [24]	104 BC patients	Urine	10 mL urine supernatant<br>Quick-DNA Urine kit (D3061, Zymo Research)	ddPCR	*TERT* 228 G>A/T mutation	The TERT 228 G>A/T mutation was counted along with the WT gene and in patients containing that mutation in their tumours as well as in cell pellet, and the detection in urine had a 92% of sensitivity
Christensen et al. 2018 [25]	65 BC patients	Plasma	Unspecified volume of plasma<br>QIAsymphony Circulating NA kit (Qiagen)	51-genes panel targeted NGS (Validation of targeted NGS by ddPCR)	51-genes panel	Development of an optimized targeted NGS approach (51-genes panel) of cfDNA from plasma
Birkenkamp-Demtröder et al. 2018 [26]	26 MIBC patients	Plasma<br>Urine	Unspecified volume of plasma and urine	ddPCR after WES for identifying an adequate number of mutations for ctDNA screening	*PIK3CA* and *FGFR3*	cfDNA analysis allows early detection of metastatic relapse and indications of treatment response

Reference	N	Liquid Biopsy	Sample Volume and cfDNA Isolation Kit Used	Detection Method	Genes Studied[1]	Clinically Relevant Findings
Soave et al. 2017 [27]	85 BC patients	Serum<br>Plasma	2 mL serum<br>2 mL plasma<br>QiAmp DNA Blood Mini kit (Qiagen)<br>QiAmp Circulating Nucleic Acid kit (Qiagen)<br>NucleoSpin Plasma XS kit (Macherey Nagel, Düren, Germany)<br>PME free-circulating DNA Extraction kit (Analytik Jena, Germany)	CNV identified by MLPA	49 genes	MLPA only detected CNVs in cfDNA extracted from serum using the PME free-circulating DNA Extraction kit
Vandekerkhove et al. 2017 [28]	51 BC patients (including 37 with metastatic disease)	Plasma	Up to 6 mL plasma<br>Circulating Nucleic Acids kit (Qiagen)	Sequencing with a combination of WES and targeted sequencing	50 BC driver genes	The distribution of all alterations found was consistent with previous studies in localized MIBC. Also, a novel *FGFR3* gene fusion was identified in consecutive samples from one patient
Casadio et al. 2017 [29][2]	52 BC patients[2]<br>46 symptomatic patients[2]<br>32 controls[2]	Urine	1 mL urine supernatant<br>Qiamp DNA minikit (Qiagen)	qPCR	*c-Myc*, *BCAS1*, *HER2* and *AR*	Urine cfDNA integrity analysis is a new, non-invasive method for early diagnosis of BC and prostate cancer
Christensen et al. 2017 [30]	363 NMIBC patients<br>468 BC patients undergoing radical cystectomy	Urine<br>Plasma	2.5-4.5 mL urine<br>2.5-4.5 mL plasma<br>QIASymphony Circulating NA kit (Qiagen)	ddPCR	*FGFR3* and *PIK3CA* hotspot mutations	Increased levels of *FGFR3* and *PIK3CA* mutated DNA in urine and plasma are indicative of later progression and metastasis in BC
Togneri et al. 2016 [31]	23 BC patients<br>12 controls	Urine	Unspecified volume of urine supernatant<br>Urine DNA Isolation Kit (Slurry Format; Norgen Biotek Corporation, Thorold, ON, Canada)	OncoScan assay for the identification of CNAs, loss of heterozygosity and recurrent clinically actionable SMs	*BRAF*, *KRAS*, *EGFR*, *IDH1*, *IDH2*, *PTEN*, *PIK3CA*, *NRAS* and *TP53*	SMs are reliably detected in urine cfDNA<br>cfDNA seems to have higher analytical sensitivity for detection of clinically actionable genomic aberrations than cellular DNA

Reference	N	Liquid Biopsy	Sample Volume and cfDNA Isolation Kit Used	Detection Method	Genes Studied[1]	Clinically Relevant Findings
Kim et al. 2016 [32]	83 BC patients<br>54 non-malignant hematuric patients<br>61 controls	Urine	1 mL urine supernatant<br>QIAquick gel extraction kit (Qiagen)	qPCR	*TopoIIA*	The expression of urine *TopoIIA* cfDNA in BC patients was significantly higher than in controls and hematuria patients. It was also higher in MIBC than in NMIBC
Birkenkamp-Demtröder et al. 2016 [33]	12 NMIBC patients (6 progressive and 6 recurrent)	Plasma<br>Urine	An average of 2.2 mL plasma<br>An average of 3.4 mL urine<br>QIAsymphony Circulating NA kit (Qiagen)	They used 3 different methods to identify genomic variants in liquid biopsies and matching tumour tissue: WGS, WES and mate-pair sequencing, and monitored the somatic variants by ddPCR	*ARID1A*, *RBM10*, *VCAN* and *KLF3*	Development of one to six personalized assays per patient for surveillance using genomic variants in ctDNA from plasma and urine<br>ctDNA can be detected in plasma and urine, even in NIBC patients, with high levels of tDNA detectable before progression, especially in urine samples
Brisuda et al. 2016 [34]	66 BC patients<br>34 controls	Urine	2 mL urine<br>QIAamp Circulating Nucleic Acid Kit (Qiagen)	qPCR	*GAPDH*	Development of a method to discriminate between BC patients and controls by quantification of urine cfDNA

[1] When a panel of genes has been employed, these have not been detailed not to overexpand. [2] The methodology of this study was detailed in a previously article from the same group, which is not included in the table on its own because it was published earlier than 2015 [35]. Abreviations: aUC, advanced urothelial carcinoma; BC, bladder cancer; CNAs, copy number alterations; cfDNA, cell-free DNA; ctDNA, circulating tumor DNA; ddPCR, Droplet Digital polymerase chain reaction; MIBC, muscle invasive bladder cancer; mLTUC, lower tract metastatic urothelial carcinoma; mUC, metastatic urothelial carcinoma; mUTUC, metastatic upper tract urothelial carcinoma; PCR, polymerase chain reaction; qPCR, real time polymerase chain reaction; UC, urothelial carcinoma; WES, whole exome sequencing; WGS, whole genome sequencing; sWGS, shallow whole genome sequencing.

References

1. Zhao, C.; Pan, Y.; Wang, Y.; Li, Y.; Han, W.; Lu, L.; Tang, W.; Li, P.; Ou, Z.; Zhang, M., et al. A novel cell-free single-molecule unique primer extension resequencing (cf-SUPER) technology for bladder cancer non-invasive detection in urine. *Transl. Androl. Urol.* **2020**, *9*, 1222–1231, doi:10.21037/tau-19-774.
2. Hayashi, Y.; Fujita, K.; Matsuzaki, K.; Eich, M.L.; Tomiyama, E.; Matsushita, M.; Koh, Y.; Nakano, K.; Wang, C.; Ishizuya, Y., et al. Clinical Significance of Hotspot Mutation Analysis of Urinary Cell-Free DNA in Urothelial Bladder Cancer. *Front. Oncol.* **2020**, *10*, 755, doi:10.3389/fonc.2020.00755.

3. Henriksen, T.V.; Reinert, T.; Christensen, E.; Sethi, H.; Birkenkamp-Demtröder, K.; Gögenur, M.; Gögenur, I.; Zimmermann, B.G.; Dyrskjøt, L.; Andersen, C.L. The effect of surgical trauma on circulating free DNA levels in cancer patients-implications for studies of circulating tumor DNA. *Mol. Onco.l* **2020**, *14*, 1670–1679, doi:10.1002/1878-0261.12729.
4. Hentschel, A.E.; Nieuwenhuijzen, J.A.; Bosschieter, J.; Splunter, A.P.V.; Lissenberg-Witte, B.I.; Voorn, J.P.V.; Segerink, L.I.; Moorselaar, R.; Steenbergen, R.D.M. Comparative Analysis of Urine Fractions for Optimal Bladder Cancer Detection Using DNA Methylation Markers. *Cancers (Basel)* **2020**, *12*, doi:10.3390/cancers12040859.
5. Pal, S.K.; Bajorin, D.; Dizman, N.; Hoffman-Censits, J.; Quinn, D.I.; Petrylak, D.P.; Galsky, M.D.; Vaishampayan, U.; De Giorgi, U.; Gupta, S., et al. Infigratinib in upper tract urothelial carcinoma versus urothelial carcinoma of the bladder and its association with comprehensive genomic profiling and/or cell-free DNA results. *Cancer* **2020**, *126*, 2597–2606, doi:10.1002/cncr.32806.
6. Ou, Z.; Li, K.; Yang, T.; Dai, Y.; Chandra, M.; Ning, J.; Wang, Y.; Xu, R.; Gao, T.; Xie, Y., et al. Detection of bladder cancer using urinary cell-free DNA and cellular DNA. *Clin. Transl. Med.* **2020**, *9*, 4, doi:10.1186/s40169-020-0257-2.
7. Ge, G.; Peng, D.; Guan, B.; Zhou, Y.; Gong, Y.; Shi, Y.; Hao, X.; Xu, Z.; Qi, J.; Lu, H., et al. Urothelial Carcinoma Detection Based on Copy Number Profiles of Urinary Cell-Free DNA by Shallow Whole-Genome Sequencing. *Clin. Chem.* **2020**, *66*, 188–198, doi:10.1373/clinchem.2019.309633.
8. Grivas, P.; Lalani, A.A.; Pond, G.R.; Nagy, R.J.; Faltas, B.; Agarwal, N.; Gupta, S.V.; Drakaki, A.; Vaishampayan, U.N.; Wang, J., et al. Circulating Tumor DNA Alterations in Advanced Urothelial Carcinoma and Association with Clinical Outcomes: A Pilot Study. *Eur. Uro.l Oncol.* **2020**, *3*, 695–699, doi:10.1016/j.euo.2019.02.004.
9. Xu, Y.; Kim, Y.H.; Jeong, P.; Piao, X.M.; Byun, Y.J.; Seo, S.P.; Kang, H.W.; Kim, W.T.; Lee, J.Y.; Ryu, D.H., et al. Urinary Cell-Free DNA IQGAP3/BMP4 Ratio as a Prognostic Marker for Non-Muscle-Invasive Bladder Cancer. *Clin. Genitourin. Cancer* **2019**, *17*, e704-e711, doi:10.1016/j.clgc.2019.04.001.
10. Ward, D.G.; Gordon, N.S.; Boucher, R.H.; Pirrie, S.J.; Baxter, L.; Ott, S.; Silcock, L.; Whalley, C.M.; Stockton, J.D.; Beggs, A.D., et al. Targeted deep sequencing of urothelial bladder cancers and associated urinary DNA: a 23-gene panel with utility for non-invasive diagnosis and risk stratification. *BJU Int.* **2019**, *124*, 532–544, doi:10.1111/bju.14808.
11. Emaus, M.N.; Varona, M.; Anderson, J.L. Sequence-specific preconcentration of a mutation prone KRAS fragment from plasma using ion-tagged oligonucleotides coupled to qPCR compatible magnetic ionic liquid solvents. *Anal. Chim. Acta.* **2019**, *1068*, 1–10, doi:10.1016/j.aca.2019.04.005.
12. Christensen, E.; Birkenkamp-Demtröder, K.; Sethi, H.; Shchegrova, S.; Salari, R.; Nordentoft, I.; Wu, H.T.; Knudsen, M.; Lamy, P.; Lindskrog, S.V., et al. Early Detection of Metastatic Relapse and Monitoring of Therapeutic Efficacy by Ultra-Deep Sequencing of Plasma Cell-Free DNA in Patients With Urothelial Bladder Carcinoma. *J. Clin. Oncol.* **2019**, *37*, 1547–1557, doi:10.1200/jco.18.02052.
13. Cheng, T.H.T.; Jiang, P.; Teoh, J.Y.C.; Heung, M.M.S.; Tam, J.C.W.; Sun, X.; Lee, W.S.; Ni, M.; Chan, R.C.K.; Ng, C.F., et al. Noninvasive Detection of Bladder Cancer by Shallow-Depth Genome-Wide Bisulfite Sequencing of Urinary Cell-Free DNA for Methylation and Copy Number Profiling. *Clin. Chem.* **2019**, *65*, 927–936, doi:10.1373/clinchem.2018.301341.
14. Hayashi, Y.; Fujita, K.; Matsuzaki, K.; Matsushita, M.; Kawamura, N.; Koh, Y.; Nakano, K.; Wang, C.; Ishizuya, Y.; Yamamoto, Y., et al. Diagnostic potential of TERT promoter and FGFR3 mutations in urinary cell-free DNA in upper tract urothelial carcinoma. *Cancer Sci.* **2019**, *110*, 1771–1779, doi:10.1111/cas.14000.
15. do Nascimento Alves, S.; Lavalhegas Hallack, M.; Moreira Perez, M.; da Costa Aguiar Alves, B.; da Silva, L.H.; Afonso Fonseca, F.L. Application of the Z-scan technique for the detection of CFCDNA (cell-free circulating DNA) and urine DNA (uDNA) in patients with bladder cancer. *Photodiagnosis Photodyn. Ther.* **2019**, *26*, 131–133, doi:10.1016/j.pdpdt.2019.02.022.
16. Stasik, S.; Salomo, K.; Heberling, U.; Froehner, M.; Sommer, U.; Baretton, G.B.; Ehninger, G.; Wirth, M.P.; Thiede, C.; Fuessel, S. Evaluation of TERT promoter mutations in urinary cell-free DNA and sediment DNA for detection of bladder cancer. *Clin. Biochem.* **2019**, *64*, 60–63, doi:10.1016/j.clinbiochem.2018.11.009.

17. Xu, Y.; Kim, Y.H.; Jeong, P.; Piao, X.M.; Byun, Y.J.; Kang, H.W.; Kim, W.T.; Lee, J.Y.; Kim, I.Y.; Moon, S.K., et al. Diagnostic value of combined IQGAP3/BMP4 and IQGAP3/FAM107A expression ratios in urinary cell-free DNA for discriminating bladder cancer from hematuria. *Urol. Oncol.* **2019**, *37*, 86–96, doi:10.1016/j.urolonc.2018.10.023.
18. Lee, D.H.; Yoon, H.; Park, S.; Kim, J.S.; Ahn, Y.H.; Kwon, K.; Lee, D.; Kim, K.H. Urinary Exosomal and cell-free DNA Detects Somatic Mutation and Copy Number Alteration in Urothelial Carcinoma of Bladder. *Sci. Rep.* **2018**, *8*, 14707, doi:10.1038/s41598-018-32900-6.
19. Li, P.; Ning, J.; Luo, X.; Du, H.; Zhang, Q.; Zhou, G.; Du, Q.; Ou, Z.; Wang, L.; Wang, Y. New method to preserve the original proportion and integrity of urinary cell-free DNA. *J. Clin. Lab. Anal.* **2019**, *33*, e22668, doi:10.1002/jcla.22668.
20. Raja, R.; Kuziora, M.; Brohawn, P.Z.; Higgs, B.W.; Gupta, A.; Dennis, P.A.; Ranade, K. Early Reduction in ctDNA Predicts Survival in Patients with Lung and Bladder Cancer Treated with Durvalumab. *Clin. Cancer Res.* **2018**, *24*, 6212–6222, doi:10.1158/1078-0432.Ccr-18-0386.
21. Kim, W.T.; Kim, Y.H.; Jeong, P.; Seo, S.P.; Kang, H.W.; Kim, Y.J.; Yun, S.J.; Lee, S.C.; Moon, S.K.; Choi, Y.H., et al. Urinary cell-free nucleic acid IQGAP3: a new non-invasive diagnostic marker for bladder cancer. *Oncotarget* **2018**, *9*, 14354–14365, doi:10.18632/oncotarget.24436.
22. Agarwal, N.; Pal, S.K.; Hahn, A.W.; Nussenzveig, R.H.; Pond, G.R.; Gupta, S.V.; Wang, J.; Bilen, M.A.; Naik, G.; Ghatalia, P., et al. Characterization of metastatic urothelial carcinoma via comprehensive genomic profiling of circulating tumor DNA. *Cancer* **2018**, *124*, 2115–2124, doi:10.1002/cncr.31314.
23. Lanman, R.B.; Mortimer, S.A.; Zill, O.A.; Sebisanovic, D.; Lopez, R.; Blau, S.; Collisson, E.A.; Divers, S.G.; Hoon, D.S.; Kopetz, E.S., et al. Analytical and Clinical Validation of a Digital Sequencing Panel for Quantitative, Highly Accurate Evaluation of Cell-Free Circulating Tumor DNA. *PLoS One* **2015**, *10*, e0140712, doi:10.1371/journal.pone.0140712.
24. Russo, I.J.; Ju, Y.; Gordon, N.S.; Zeegers, M.P.; Cheng, K.K.; James, N.D.; Bryan, R.T.; Ward, D.G. Toward Personalised Liquid Biopsies for Urothelial Carcinoma: Characterisation of ddPCR and Urinary cfDNA for the Detection of the TERT 228G>A/T Mutation. *Bladder Cancer* **2018**, *4*, 41–48, doi:10.3233/blc-170152.
25. Christensen, E.; Nordentoft, I.; Vang, S.; Birkenkamp-Demtröder, K.; Jensen, J.B.; Agerbæk, M.; Pedersen, J.S.; Dyrskjøt, L. Optimized targeted sequencing of cell-free plasma DNA from bladder cancer patients. *Sci. Rep.* **2018**, *8*, 1917, doi:10.1038/s41598-018-20282-8.
26. Birkenkamp-Demtröder, K.; Christensen, E.; Nordentoft, I.; Knudsen, M.; Taber, A.; Høyer, S.; Lamy, P.; Agerbæk, M.; Jensen, J.B.; Dyrskjøt, L. Monitoring Treatment Response and Metastatic Relapse in Advanced Bladder Cancer by Liquid Biopsy Analysis. *Eur. Urol.* **2018**, *73*, 535–540, doi:10.1016/j.eururo.2017.09.011.
27. Soave, A.; Chun, F.K.; Hillebrand, T.; Rink, M.; Weisbach, L.; Steinbach, B.; Fisch, M.; Pantel, K.; Schwarzenbach, H. Copy number variations of circulating, cell-free DNA in urothelial carcinoma of the bladder patients treated with radical cystectomy: a prospective study. *Oncotarget* **2017**, *8*, 56398–56407, doi:10.18632/oncotarget.17657.
28. Vandekerkhove, G.; Todenhöfer, T.; Annala, M.; Struss, W.J.; Wong, A.; Beja, K.; Ritch, E.; Brahmbhatt, S.; Volik, S.V.; Hennenlotter, J., et al. Circulating Tumor DNA Reveals Clinically Actionable Somatic Genome of Metastatic Bladder Cancer. *Clin. Cancer Res.* **2017**, *23*, 6487–6497, doi:10.1158/1078-0432.Ccr-17-1140.
29. Casadio, V.; Salvi, S.; Martignano, F.; Gunelli, R.; Ravaioli, S.; Calistri, D. Cell-Free DNA Integrity Analysis in Urine Samples. *J Vis Exp* **2017**, 10.3791/55049, doi:10.3791/55049.
30. Christensen, E.; Birkenkamp-Demtröder, K.; Nordentoft, I.; Høyer, S.; van der Keur, K.; van Kessel, K.; Zwarthoff, E.; Agerbæk, M.; Ørntoft, T.F.; Jensen, J.B., et al. Liquid Biopsy Analysis of FGFR3 and PIK3CA Hotspot Mutations for Disease Surveillance in Bladder Cancer. *Eur. Urol.* **2017**, *71*, 961–969, doi:10.1016/j.eururo.2016.12.016.
31. Togneri, F.S.; Ward, D.G.; Foster, J.M.; Devall, A.J.; Wojtowicz, P.; Alyas, S.; Vasques, F.R.; Oumie, A.; James, N.D.; Cheng, K.K., et al. Genomic complexity of urothelial bladder cancer revealed in urinary cfDNA. *Eur. J. Hum. Genet.* **2016**, *24*, 1167–1174, doi:10.1038/ejhg.2015.281.

32. Kim, Y.H.; Yan, C.; Lee, I.S.; Piao, X.M.; Byun, Y.J.; Jeong, P.; Kim, W.T.; Yun, S.J.; Kim, W.J. Value of urinary topoisomerase-IIA cell-free DNA for diagnosis of bladder cancer. *Investig. Clin. Urol.* **2016**, *57*, 106–112, doi:10.4111/icu.2016.57.2.106.
33. Birkenkamp-Demtröder, K.; Nordentoft, I.; Christensen, E.; Høyer, S.; Reinert, T.; Vang, S.; Borre, M.; Agerbæk, M.; Jensen, J.B.; Ørntoft, T.F., et al. Genomic Alterations in Liquid Biopsies from Patients with Bladder Cancer. *Eur. Urol.* **2016**, *70*, 75–82, doi:10.1016/j.eururo.2016.01.007.
34. Brisuda, A.; Pazourkova, E.; Soukup, V.; Horinek, A.; Hrbáček, J.; Capoun, O.; Svobodova, I.; Pospisilova, S.; Korabecna, M.; Mares, J., et al. Urinary Cell-Free DNA Quantification as Non-Invasive Biomarker in Patients with Bladder Cancer. *Urol. Int.* **2016**, *96*, 25–31, doi:10.1159/000438828.
35. Casadio, V.; Calistri, D.; Tebaldi, M.; Bravaccini, S.; Gunelli, R.; Martorana, G.; Bertaccini, A.; Serra, L.; Scarpi, E.; Amadori, D., et al. Urine cell-free DNA integrity as a marker for early bladder cancer diagnosis: preliminary data. *Urol. Oncol.* **2013**, *31*, 1744–1750, doi:10.1016/j.urolonc.2012.07.013.
